# Correlation analysis of anthropometric indices and type 2 diabetes mellitus in residents aged 60 years and older

**DOI:** 10.3389/fpubh.2023.1122509

**Published:** 2023-03-29

**Authors:** Xiaoyan Feng, Junyi Wang, Shupei Wang, Zhihao Wang, Shan Wu, Yuan Wei, Lvrong Li, Tianran Shen, Qingsong Chen

**Affiliations:** ^1^Guangdong Provincial Engineering Research Center of Public Health Detection and Assessment, Guangdong Pharmaceutical University, Guangzhou, China; ^2^School of Public Health, Guangdong Pharmaceutical University, Guangzhou, China

**Keywords:** diabetes mellitus, abdominal obesity, China visceral obesity index, neck circumference, visceral obesity index

## Abstract

**Background and purpose:**

In recent years, the incidence of obesity in people aged 60 and over has increased significantly, and abdominal obesity has been recognized as an independent risk factor for diabetes. Aging causes physiologic decline in multiple body systems, leading to changes in obesity indicators such as BMI. At present, the relationship between abdominal obesity markers and Diabetes mellitus (DM) in people aged 60 years and older remains unclear. Therefore, it is necessary to study the correlation between anthropometric indices and diabetes and explore potential predictors.

**Methods:**

The basic demographic information of participants aged 60 and above in Zhongshan City in 2020 was collected. Physical parameters, blood glucose and other biochemical indices were measured comprehensively. Binary logistic regression analysis was used to explore the relationship between abdominal obesity indicators [Waist circumference, Neck Circumference, Waist-to-hip ratio, Chinese Visceral Obesity Index (CVAI), and visceral obesity index] and diabetes mellitus. ROC characteristic curve was used to analyze the predictive ability of abdominal obesity indicators to DM, and the non-restrictive cubic spline graph was used to visualize the screened obesity indicators and diabetes risk.

**Results:**

Among 9,519 participants, the prevalence of diabetes was 15.5%. Compared with low CVAI, High CVAI level was significantly associated with increased prevalence of DM in males and females (all *p* < 0.05), in males (OR, 2.226; 95%CI: 1.128–4.395), females (OR, 1.645; 95%CI: 1.013–2.669). After adjusting for potential confounding factors, there were gender differences between neck circumference and the prevalence of DM, and above-normal neck circumference in males was significantly associated with increased prevalence of DM (OR, 1.381; 95% CI: 1.091–1.747) (*p* < 0.05).

**Conclusion:**

Among these anthropometric indices, CVAI is consistent with the features of fat distribution in older individuals and shows superior discriminative power as a potential predictor of DM, compared to traditional anthropometric parameters.

## 1. Introduction

Diabetes mellitus (DM) is a metabolic disorder caused by genetic and environmental factors. Its high prevalence and high disability rate have caused serious health damage worldwide (1). Obesity is one of the important reasons for insulin resistance/insufficient insulin secretion and increased blood glucose (2). With the rise in obesity rate, the incidence of diabetes also elevated (3). As growing aged, older individuals are prone to such problems as a decrease in bone mineral density (4), loss of muscle content (5), body dysfunction and metabolic disorders (6), thus they are much more likely to develop diabetes and most metabolic risks than the middle-aged and young (7). Data show that the prevalence of diabetes among adults in China increased from 0.67% in 1980 to 12.8% in 2018 (8). Some studies estimate that the number of diabetes patients worldwide will increase from 451 million to 693 million from 2017 to 2045 (9). The prevalence of diabetes in the older individual in China is 30.2%, far higher than the prevalence of diabetes in the world (19.3%), ranking first in the world (10). About two-thirds of patients with type 2 diabetes are overweight or obese, and abdominal obesity accounts for about 50% (11). Therefore, being overweight and obesity, especially abdominal obesity, is considered to be independent risk factors for type 2 diabetes mellitus (T2DM), which also aggravate the risk of disability and complicate its management. To some extent, obesity indicators are easier to observe and measure directly. The research on the relationship between obesity indices and diabetes aims to achieve the prevention and control of diabetes through the recognition of obesity.

Obesity is defined as excess body fat resulting in health impairment, which is usually evaluated in clinical practice through body mass index (BMI), but it cannot distinguish between fat and lean body mass. For the older individual, BMI shows an increase with age due to the decrease in spine height and fat mass expansion (12). Abdominal obesity, also known as central obesity, refers to the excessive accumulation of fat in abdominal or abdominal organs (13). Waist circumference (WC) is often used to measure visceral fat content, which can better reflect visceral obesity than BMI (14), but it has limitations in distinguishing Visceral Adipose tissue (VAT) from subcutaneous fat (15). Magnetic resonance imaging (MRI) and computed tomography (CT) are considered the gold standards for body fat measurement, but are not recommended in routine clinical practice or large-scale epidemiological studies due to radiation, time-consuming, and high cost (16, 17). As more anthropometric indicators emerge, the indicators of abdominal obesity mainly include simple physical indicators and composite indicators. Simple physical measures such as WC, NC, Waist-Hip ratio (WHR), and Waist-Height ratio (WHtR). Compound measures are calculated based on WC, BMI, triglyceride (TG) and high-density lipoprotein (HDL) data, such as visceral obesity index (VAI) and lipid accumulation product (LAP) (18). Different indexes of obesity have their own defects and advantages. It is noteworthy that the Chinese Visceral Obesity Index (CVAI) was recently established by Xia, et al. (19) as an indicator of abdominal obesity based on age, BMI, WC, and metabolic parameters, and is considered to be a better predictor of diabetes than VAI, BMI, WC, and WHR (20). Although a large number of studies have explored the predictive power of obesity and abdominal obesity indicators for diabetes, adipose tissue function is at the intersection of processes leading to age-related metabolic diseases and mediating longevity. Hormonal fluctuations in aging may regulate age-related visceral obesity and metabolic dysfunction (21). Therefore, it is necessary to study the correlation between anthropometric indices and diabetes mellitus in the population aged 60 and above. In this study, based on residents aged 60 years and older in a Chinese community, we aimed to investigate the association of anthropometric indices (WC, NC, WHR, WHtR, LAP, VAI, and CVAI) with the risk of diabetes and compare their performance as potential predictors of DM. This is important for capturing the health status of older individuals and for early detection and precise prevention and control of diabetes.

## 2. Materials and methods

### 2.1. Study design and participants

This study was a cross-sectional survey of cluster sampling, and before the start of the study, we calculated the population sample size (the calculation results were shown in Supplementary material). Based on the National Basic Public-health service for the older individual in Minzhong Town and Torch Development Zone of Zhongshan City, Guangdong Province, 11,003 participants aged 60 years and older who had lived in the two towns for at least 6 months without severe infectious diseases, end-stage tumors, recent major surgeries, and external injuries, were initially recruited for this study through local advertisements, invitation letters, health talks, or referrals from the local community, from May to October 2020. Trained staff administered face-to-face interviews to collect information on participants’ demographic characteristics, lifestyle, and habits (e.g., smoking, alcohol drinking, physical activity and etc.) and histories of chronic diseases through standardized questionnaires (The questionnaire was shown in Supplementary material). Physical examination, clinical evaluation, and laboratory testing were performed by expert physicians. We excluded participants, who would not accept laboratory testing or physical examination(n = 1,328), or whose information was incomplete (*n* = 156). A total of 9,519 participants were included in this analysis. The study protocol was approved by the Ethics Committee of Guangdong Pharmaceutical University, and all study participants provided written informed consent. All procedures were conducted by the ethical guidelines of the Declaration of Helsinki.

### 2.2. The body measurement

Anthropometric measurements include height, weight, WC, NC, HC, blood pressure, etc. Medical examinations are performed by uniformly trained investigators using standard methods. The measurement methods were in accordance with the industry standard of the People’s Republic of China-Human health Monitoring anthropometric Method (WS/T424-2013) standard requirements. All participants removed their shoes and wore light clothing during the measurements. Height was measured by a metal column height meter [measured by Meida (Shanghai) Medical Instrument Co., LTD.], weight was measured by an electronic weight scale [Tanita HD-390 electronic scale (Shanghai) Trading Co., LTD.], neck, waist and hip circumference were measured with a tape measure. The measurement plane of the NC is located at the throat node of the neck; The measurement plane of WC passes through the lower edge of the bilateral midaxillary costal arch and the midpoint of the line of the iliac crest; The hip measurement plane is the body circumference at the highest point of the hip. BMI, WHR, WHtR, LAP, VAI, and CVAI were calculated as follows:

$$BMI=\mathrm{weight}\;\left(\mathrm{kg}\right)/\mathrm{height}\;{\left(m\right)}^{2}$$

$$WHR=\mathrm{waistline}\;\left(\mathrm{cm}\right)/\mathrm{hip}\;\mathrm{circumference}\;\left(\mathrm{cm}\right)$$

$$\mathrm{WHtR}=\mathrm{waistline}\;\left(\mathrm{cm}\right)/\mathrm{height}\;\left(\mathrm{cm}\right)$$

$$\begin{array}{l} \mathrm{Men}:\mathrm{VAI}=\mathrm{WC}\;\left(\mathrm{cm}\right)/\left[39.68+1.88\times \mathrm{BMI}\left(\mathrm{kg}/m^{2}\right)\right]\times \\ \left[\mathrm{TG}\left(\mathrm{mmol}/L\right)/1.03\right]\times \left[1.31/\mathrm{HDL}\left(\mathrm{mmol}/L\right)\right] \end{array}$$

$$\begin{array}{l} \mathrm{CVAI}=-267.93+0.68\times \mathrm{age}+0.03\times \mathrm{BMI}+4\times \mathrm{WC}\left(\mathrm{cm}\right)+22\times \\ \log_{10}\mathrm{TG}\left(\mathrm{mmol}/L\right)-16.32\times \mathrm{HDL}\left(\mathrm{mmol}/L\right)\\ \mathrm{LAP}=\left[\mathrm{WC}\left(\mathrm{cm}\right)-65\right]\times \mathrm{TG}\left(\mathrm{mmol}/L\right) \end{array}$$

$$\begin{array}{l} \mathrm{Women}:\mathrm{VAI}=\mathrm{WC}\;\left(\mathrm{cm}\right)/\left[\begin{array}{l} 36.58+1.89\times \\ \mathrm{BMI}\left(\mathrm{kg}/m^{2}\right) \end{array}\right]\times \left[\begin{array}{l} \mathrm{TG}\left(\mathrm{mmol}/L\right)/\\ 0.81 \end{array}\right]\times \\ \left[1.52/\mathrm{HDL}\left(\mathrm{mmol}/L\right)\right]\\ \mathrm{CVAI}=-187.32+1.71\times \mathrm{age}+4.32\times \mathrm{BMI}+1.12\times \mathrm{WC}\left(\mathrm{cm}\right)+\\ 39.76\times \log_{10}\mathrm{TG}\left(\mathrm{mmol}/L\right)-11.66\times \mathrm{HDL}\left(\mathrm{mmol}/L\right)\\ \mathrm{LAP}=\left[\mathrm{WC}\left(\mathrm{cm}\right)-58\right]\times \mathrm{TG}\left(\mathrm{mmol}/L\right) \end{array}$$

### 2.3. Biochemical measurements

All participants were asked to have an empty stomach for at least 8 h before the measurement. Fasting venous blood was collected from the participants. Blood samples for blood glucose detection were collected by EDTA-K2 vacuum anticoagulant tube and centrifuged within 2 h after collection. The serum was collected in equal parts by freezing at room temperature −20°C and then transported to Zhongshan Torch Development Zone Hospital with dry ice within 2–4 h. Fasting blood glucose (FPG), serum creatinine, triglyceride, total cholesterol, high-density lipoprotein (HDL) and low-density lipoprotein (LDL) were measured by Beckman Coulter AU680 (Brea, United States).

### 2.4. Definition of major variables

According to The Chinese Guidelines for Diabetes Prevention and Treatment 2020 edition, diabetes is defined as a registered diagnosis in the registry, treatment with antidiabetic drugs, or self-reported diabetes in the questionnaire. Body mass index (BMI) is divided into four levels according to the Chinese Adult Overweight and Obesity Prevention and Control Guidelines: BMI < 18.5 kg/m^2^ is underweight, 18.5–23.9 kg/m^2^ is normal, 24–27.9 kg/m^2^ is overweight, and BMI ≥ 28 kg/m^2^ is obese. Abdominal obesity: female WC >80 cm, male WC > 90 cm or female waist-to-hip ratio (WHR) >0.8, male WHR > 0.9; Waist to height ratio (WHtR) >0.5. Other factors: smokers were defined as those who had smoked more than 100 cigarettes per five packs, while drinkers were defined as those who consumed alcoholic beverages at least once a week for 6 months. Exercise According to the 2020 WHO guidelines on physical activity and sedentary behavior, adequate moderate recreational physical activity (MRPA) is defined as no less than 150 min of MRPA or 75 min of vigorous recreational physical activity (VRPA) or an equivalent combination of MRPA and VRPA (1 min of VRPA is equal to 2 min of MRPA) per week for adults.

### 2.5. Statistical analysis

Data on potential confounders have a completion rate of more than 95%. The remaining missing values are filled by multiple interpolations. Continuous variables satisfying normal distribution are expressed as $\bar{X}\pm S$ , those not satisfying normal distribution are expressed as the *M* (p25–p75), and categorical variables are expressed as percentages (%). When comparing continuous variables between groups, it is determined whether the variables satisfy a normal distribution. If yes, the Student’s *T*-test was used; if not, the Mann–Whitney *U* test is used. The categorical variables were compared between groups using chi-squared tests. Statistical significance measures screened by differences between groups could be considered for inclusion in the regression model. Multivariate binary Logistic regression was used to analyze the relationship between WC, NC, WHR, WHtR, LAP, VAI, and CVAI and diabetes mellitus. Receiver operating characteristic (ROC) curves were used to compare the predictive abilities of WC, NC, BMI, VAI and CVAI for DM in males and females. Pair comparisons of the areas under the ROC curve (AUC) of these abdominal obesity indexes were analyzed using the *Z*-test. Restrictive Cubic Spline plots (RCS) were used to visualize the correlation between obesity and diabetes risk. A two-sided test *p* < 0.05 was considered statistically significant. Statistical analysis was performed using SPSS software (SPSS Inc., Version 25.0).

## 3. Results

### 3.1. Basic characteristics of the research object

Among the 9,519 participants aged 60 or above, 5,638 (59.2%) were female and 3,881 (40.7%) were male, the prevalence of diabetes was 15.5%. The mean age of male participants was 71.07 ± 6.097 years; The female’s average age was 70.62 ± 6.207, and 67.3% had primary school education or below. Smokers accounted for 15.3% and drinkers for 17.7%; Table 1 shows the sociodemographic and general characteristics of male and female participants. Participants were divided into two groups, with or without diabetes mellitus (DM). Compared with men without DM, the DM group had significant differences in education level, smoking, alcohol consumption, BMI, Blood pressure, WC, NC, WHR, WHtR, BMI, VAI, LAP, and CVAI (all *p* < 0.05). Compared with non-DM women, the DM group had significant differences in alcohol consumption, blood pressure, WC, NC, WHR, WHtR, BMI, VAI, LAP, and CVAI (all *p* < 0.05).

**Table 1:** General characteristics of participants.

		Man			Woman	
Characteristic	DM *N* = 580(%)	Non-DM *N* = 3,301(%)	*P*	DM *N* = 898(%)	Non-DM *N* = 4,740(%)	*P*
Age, year	70.99 ± 6.22	71.09 ± 6.076	0.709	70.81 ± 6.319	70.58 ± 6.185	0.709
education level			0.004			0.852
Primary school and below	290(50)	1910(57.9)		668(74.4)	3,552(74.9)	
Drinking			0.007			0.008
Yes	154(26.6)	1,067(32.3)		848(94.4)	4,356(91.9)	
No	426(73.4)	2,234(67.7)		50(5.6)	384(8.1)	
Smoking(%)			0.016			0.355
Non-smokes	289(49.8)	1,554(47.1)		878(97.8)	4,588(96.8)	
Current smokers	169(29.1)	1,153(34.9)		14(1.6)	107(2.3)	
Former smokers	122(21)	594(18)		6(0.7)	45(0.9)	
Physical activity smoke(%)			0.171			0.689
No	267(74.7)	1,610(50.3)		458(51)	2,381(50.2)	
Yes	299(52.8)	1,588(49.7)		440(49)	2,359(49.8)	
BMI (kg/m^2^)			<0.001*			<0.001
<18.5	16(2.8)	192(5.8)		18(2.0)	281(5.9)	
18.5–23.9	264(45.5)	1778(53.9)		371(41.3)	2,272(47.9)	
24–27.9	231(39.8)	1,096(33.2)		368(41.0)	1,643(34.7)	
≥28	69(11.9)	235(7.1)		141(15.7)	544(11.5)	
Blood pressure						
Systolic blood pressure			0.027			<0.001
<140	298(51.4)	1860(56.3)		420(46.8)	2,463(52.0)	
≥140	282(48.6)	1,441(43.7)		478(53.2)	2,277(48.0)	
Diastolic blood pressure			0.053			0.063
<90	270(46.6)	1,682(51.0)		405(45.1)	2,299(48.5)	
≥90	310(53.4)	1,619(49)		493(54.9)	3,441(51.5)	
WC			<0.001*			<0.001
Standard	294(50.7)	2,303(69.8)		186(20.7)	1,641(34.6)	
Obesity	286(49.3)	998(30.2)		2,712(79.3)	3,099(65.4)	
NC			<0.001*			<0.001
Standard	343(59.1)	2,484(75.2)		675(75.2)	3,892(82.1)	
Obesity	237(40.9)	817(24.8)		223(24.8)	848(17.9)	
WHR			<0.001*			<0.001
Standard	118(20.3)	1989(60.3)		19(2.1)	305(6.4)	
Obesity	462(79.7)	1,312(39.7)		879(97.9)	4,435(93.6)	
WHtR			<0.001*			<0.001
Standard	92(15.9)	1,111(33.7)		88(9.8)	942(19.9)	
Obesity	488(84.1)	2,190(66.3)		810(90.2)	3,798(80.1)	
VAI			<0.001*			<0.001
Quartile 1	92(15.9)	879(26.6)		143(15.9)	1,267(26.7)	
Quartile 2	109(18.8)	861(26.1)		203(22.6)	1,206(25.4)	
Quartile 3	167(28.8)	803(24.3)		237(26.4)	1,173(24.7)	
Quartile 4	212(36.6)	758(23)		315(35.1)	1,094(23.1)	
LAP			<0.001*			<0.001
Quartile 1	898(27.2)	76(13.1)		135(15.0)	1,275(26.9)	
Quartile 2	856(25.9)	112(19.3)		201(22.4)	1,208(25.5)	
Quartile 3	795(24.1)	174(30.0)		241(26.8)	1,169(24.7)	
Quartile 4	752(22.8)	218(37.6)		321(35.7)	1,088(23.0)	
CVAI			<0.001*			<0.001
Quartile 1	68(11.7)	903(27.4)		130(14.5)	1,280(27.0)	
Quartile 2	95(16.4)	875(26.5)		199(22.2)	1,210(25.5)	
Quartile 3	190(32.8)	780(23.6)		253(28.2)	1,157(24.4)	
Quartile 4	227(39.1)	743(22.5)		316(35.2)	1,093(23.1)	

*BMI, Body Mass Index; WC, Waist Circumference; NC, neck circumference; WHR, Waist-Hip ratio; WHtR, Waist-Height ratio; VAI, visceral obesity index; LAP, Lipid Accumulation Product; CVAI, Chinese Visceral Obesity Index. *P* < 0.05, The difference was statistically significant.

Correlation analysis was conducted on the study variables (the results were shown in Supplementary Table S1). The diagnosis was made according to the following criteria: Check whether there is collinearity between the variables in the correlation coefficient table. It is generally believed that collinearity between variables can be considered if the correlation coefficient is greater than 0.7. The results showed that the correlation coefficient between LAP and VAI was 0.90, and *p* < 0.05, indicating a strong correlation between independent variables, indicating collinearity between independent variables. Variables that are not collinear are selected. After adjusting for age, education level, smoking, alcohol consumption, physical activity, BMI, diastolic blood pressure, systolic blood pressure and fasting blood glucose, the relationship between abdominal obesity and the prevalence of DM was shown in Table 2. Compared with low CVAI, high CVAI level was significantly associated with the increased prevalence of DM in both males and females (*p* < 0.05), in males (OR, 2.226; 95%CI: 1.128–4.395), females (OR, 1.645; 95%CI: 1.013–2.669). However, there were differences between other abdominal obesity indicators and the prevalence of DM in men and women, and the increase in NC was significantly correlated with the increase of DM prevalence in men (OR, 1.381; 95% CI: 1.091–1.747); Higher VAI levels were significantly associated with an increased prevalence of DM compared with lower VAI levels in women (OR, 1.604; 95% CI: 1.209–2.127).

**Table 2:** Multivariate analysis of anthropometric indices and diabetes mellitus in the older individual.

Variable		Men			Woman	
	OR	95% CI	*P*	OR	95% CI	*P*
NC						
Standard		1(ref.)			1(ref.)	
Obesity	1.381	1.091–1.747	**0.007**	1.133	0.937–1.374	0.205
WC						
Standard		1(ref.)			1(ref.)	
Obesity	1.183	0.962–1.788	0.338	1.265	0.980–1.633	0.071
WHR						
Standard		1(ref.)			1(ref.)	
Obesity	1.312	0.962–1.788	0.086	1.644	0.980–2.759	0.060
WHtR						
Standard		1(ref.)			1(ref.)	
Standard	1.159	0.764–1.756	0.488	1.091	0.779–1.526	0.613
VAI						
Quartile 1		1(ref.)			1(ref.)	
Quartile 2	0.927	0.676–1.272	0.639	1.231	0.967–1.566	0.092
Quartile 3	1.156	0.843–1.587	0.368	1.265	0.976–1.640	0.075
Quartile 4	1.337	0.957–1,868	0.089	1.604	1.209–2.127	0.001
CVAI						
Quartile 1		1(ref.)			1(ref.)	
Quartile 2	1.162	0.747–1.806	0.506	1.109	0.814–1.508	0.509
Quartile 3	2.104	1.221–3.627	0.007	1,311	0.893–1.923	0.166
Quartile 4	2.226	1.128–4.395	0.021	1.645	1.013–2.669	0.044

*Adjusted for age, education level, smoking, alcohol consumption, physical activity, BMI, diastolic blood pressure, systolic blood pressure and fasting blood glucose. NC, neck circumference; WC, Waist Circumference; WHR, Waist-Hip Ratio; WHtR, Waist-Height ratio; VAI, visceral obesity index; CVAI, Chinese Visceral Obesity Index. *P* < 0.05 indicates statistical difference.

### 3.2. Receiver operating characteristic curves

**Figure 1:**
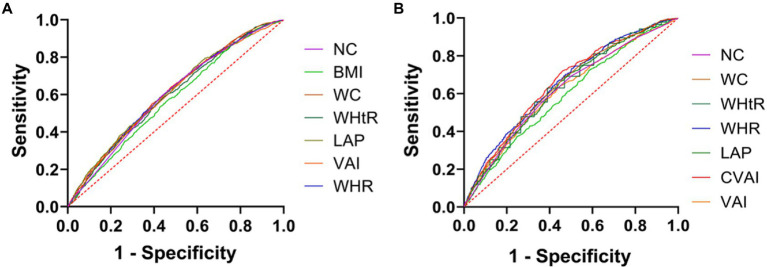
Anthropometric indices ROC curves for the diagnosis of DM in men and women. **(A)** ROC curves of indicators of abdominal obesity and diabetes in women. **(B)** ROC curves of indicators of abdominal obesity and diabetes in women.

Figure 1 is based on the ROC curve to analyze the diagnostic ability of anthropometric Indices (including BMI, WC, NC, WHR, LAP, VAI, and CVAI) for DM in men and women. BMI, WC, NC, WHR, LAP, VAI, and CVAI were statistically different between males and females (*p* < 0.01). In Figure 1A, the areas under ROC curve of BMI, WC, NC, WHR, WHtR, VAI and CVAI of male DM were 0.590, 0.644, 0.620, 0.653, 0.634, 0.616 and 0.655 (all *p* < 0.001). Compared with other indicators of abdominal obesity, CVAI had the largest area under the ROC curve, the critical value of the maximum Youden index was 129.883, the sensitivity was 71.9%, and the specificity was 54.1%. The areas under the ROC curve of BMI, WC, NC, WHR, WHtR, VAI, and CVAI in female DM were 0.573, 0.602, 0.579, 0.605, 0.594, 0.601, and 0.607 (all *p* < 0.001), as shown in Figure 1B. Compared with other indicators of abdominal obesity, CVAI had the largest area under the ROC curve, and the critical value of the maximum Youden index of CVAI was 125.883, with a sensitivity of 64.1% and specificity of 52%.

### 3.3. Dose–response relationship between CVAI and risk of DM

**Figure 2:**
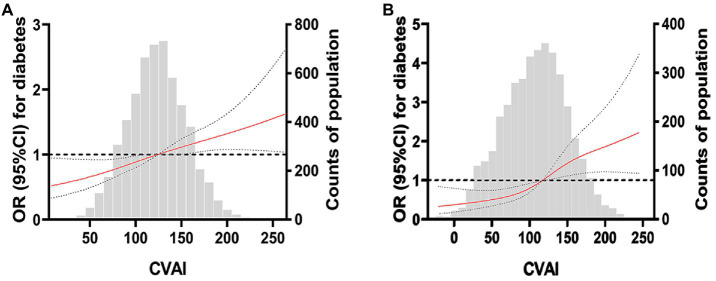
Restricted cubic spline plot of China Visceral Adiposity and diabetes. **(A)** RCS curve of CVAI and diabetes risk in women. **(B)** RCS curve of CVAI and diabetes risk in men. The solid red line is the multivariable-adjusted hazard ratio, and the dashed black line shows the 95% confidence interval from the restricted cubic spline regression. Unassociated reference lines are indicated by bold dotted lines and have a hazard ratio of 1.0. The gray histogram shows the frequency distribution of people with different CVAI levels. The correlation between CVAI level and the risk of DM in the continuous range was approximately linear, and a higher CVAI level was associated with an increased risk of DM.

After adjusting for age, education level, smoking, alcohol consumption, physical activity, systolic blood pressure, diastolic blood pressure, and fasting blood glucose, Figures 2A,B show that CVAI had a linear dose–response relationship with the risk of diabetes (linear test; *p* < 0.001).

### 3.4. Sensitivity analysis

We also tried the binary logistic analysis of the continuous variables NC, WC, WHR, WHtR, VAI, CVAI, and diabetes (this table is in Supplementary Table S2) and the decision tree model to analyze the classified data to check the results of the variables NC, WC, WHR, WHtR, VAI, CVAI, and diabetes. Supplementary Table S2 shows that in men, NC, WC, WHR, and CVAI are associated with a higher prevalence of DM; in women, NC, WHR, and CVAI were significantly associated with an increased prevalence of DM events. Supplementary Figure S1 shows that in men, increased levels of NC, WHR and CVAI are associated with a higher prevalence of DM. Supplementary Figure S2 shows that in women, increased levels of WC, WHtR, VAI, and CVAI were associated with an increased prevalence of DM. The probability of DM events in males and females with high CVAI was 21.5 and 23%.

## 4. Discussion

Visceral obesity index is a significant feature to measure abdominal obesity (6). Compared with subcutaneous fat obesity, visceral fat obesity has been identified as a key factor leading to insulin resistance, cardiovascular disease and metabolic syndrome in the human body, and has been proposed as a marker of obesity dysfunction and ectopic fat deposition. There were gender and age differences in anthropometric indices. Especially in postmenopausal women, the content and distribution of body fat tissue are quite different from that of women in general, due to changes in sex hormones. With the growth of age, fat distribution in the elderly changes drastically in contrast to general adults (22). To our knowledge, the evidence for the association of abdominal obesity measures, such as NC, VAI, CVAI, and LAP with diabetes in people 60 years of age and older remains limited. Therefore, in this community-based study based on 9,519 participants aged 60 years and older, we examined the association of visceral obesity indices NC, WC, VAI, CVAI, and LAP with diabetes after BMI adjustment and found that CVAI was more strongly associated with diabetes than VAI, BMI, NC, WHR, and WHtR. A linear relationship was found between CVAI levels and the prevalence of diabetes, and the risk of diabetes increased when CVAI levels were higher than the critical value. Although adjusted for multiple forms of abdominal obesity, it is unclear whether other associations can be attributed to residual confounding of alternative mechanisms.

A meta-analysis of a global multi-ethnic population has shown that multiple anthropometric indicators, including VAI, BMI, and WC are strongly associated with diabetes risk (20, 23). However, as a marker of abdominal obesity, CVAI is better than conventional anthropometric and visceral obesity indicators in the Asian population (24, 25) and has been proven to be successful in predicting DM events in Chinese (23, 26) and other ethnic groups (27, 28). We further extend their results to the Chinese population aged 60 years and older and explore whether there is a significant difference in optimal visceral fat area cutoff values between males and females. Consistent with the previous report, all anthropometric measures were able to identify baseline diabetes (AUC > 0.5). Only CVAI produced the highest AUC for diabetes and was gender-consistent. But the obesity marker was better at identifying diabetes in men than in women. This is mainly due to the significant increase in visceral fat content during aging in men, while in women, a greater change in preperitoneal circumference was observed (29). It is possible that when visceral fat is excessively accumulated in the body, macrophages infiltrate around hypertrophic adipocytes, leading to the massive release of inflammatory factors (IL6, tumor necrosis factor) and the reduction of adiponectin, thereby reducing insulin sensitivity, and eventually showing insulin resistance and lipid metabolism disorders, and then developing metabolic syndrome and T2DM (30). The exact reason behind this result remains to be clarified. It is also partly hypothesized that this is due to gender differences in visceral fat distribution (31), or because Asian women generally have greater abdominal obesity and obesity index, thereby increasing the associated cardiometabolic risk (like HDL or TG) (32).

Independent of respiration and stomach fullness (33), NC can be used as an alternative indicator of subcutaneous fat distribution in the upper body to identify obesity and predict obesity-related metabolic diseases (34). Some studies have reported that NC is independently associated with hyperuricemia (35), non-alcoholic fatty liver disease (36), and sleep apnea syndrome (37). In addition, large NC is also an independent predictor of metabolic syndrome and positively correlated with visceral adipose tissue and insulin resistance (38, 39), which is similar to our findings. This may be related to the fact that fat distribution in older obese men is predominantly in the neck and trunk; in women, it is predominantly in the abdomen and below and the hip extremities (29). In fact, the NC of men is significantly larger than that of women. In this study, we separately classified this difference to make the NC of men and women comparable. The results showed that the NC of men was uniquely correlated with DM, while the correlation was not prominent in women. Many studies have also found that NC is closely associated with obesity-related metabolic diseases. The SABPA cohort study found that NC was associated with blood pressure, blood glucose, lipids, and insulin levels compared to WC, and was a stronger predictor of metabolic syndrome (Mets) (independent of menopausal status) in Saharan Caucasians (40). The Framingham Heart Study in the US, which followed 2,732 people for 10 years, found that higher NC was associated with high blood pressure and all cardiometabolic risk factors (41). A meta-study found a stronger association between NC and BMI and WC in men over 50 than in other demographic groups (42), and this further confirms our research. At present, the exact mechanism by which neck adipose tissue is linked to diabetes is not yet fully established. In a relatively small area of the neck, there are two pools of peripheral fat around the bilateral carotid vessels (43). They secrete various adipokines, including interlukin-6 and leptin, which are involved in the development of diabetes and metabolic disorders (44). Current evidence suggests that high plasma-free fatty acids (FFAs) provide the basis for metabolic disorders. Excess FFAs play a role in increasing very low-density lipoprotein production and inhibiting insulin clearance, leading to insulin resistance (45). The concentration of FFAs of free fat is mainly affected by NC value, which is mainly because the lipolysis capacity and FFAs release rate of subcutaneous fat in the upper body are higher than those in the lower body (46).

WC could reflect abdominal cellulite but could not distinguish visceral cellulite from subcutaneous cellulite. Our study showed no significant difference between WC and DM risk, regardless of adjusted BMI. However, studies have confirmed that there is a linear correlation between WC and diabetes risk in the general population (23). Different from other studies, the possible reasons are that, on the one hand, with the aging process of the older individual, inflammation will lead to the redistribution of fat into the abdominal region (visceral fat) and skeletal muscle, increasing the amount of fat infiltrated in the liver, muscle and other organs of the older individual, while the amount of subcutaneous fat decreases (47). Therefore, the WC standard of the general population may be used to judge the obesity rate of the older individual, but it needs to be further verified by large sample cohort studies. On the other hand, lipids and their derivatives accumulate within and between muscle cells, inducing mitochondrial dysfunction, metabolic disorders, lipid toxicity, and insulin resistance, and enhancing the secretion of some pro-inflammatory cytokines. In turn, cytokines secreted by these muscles can also aggravate adipose tissue atrophy, maintain chronic low-grade inflammation, and establish a vicious cycle of local hyperlipidemia and insulin resistance (48). Therefore, WC alone cannot be used to measure the risk of diabetes in older individuals.

Our work represents a large-scale study demonstrating the utility of CVAI as a potential screening tool for diabetes in the Chinese population aged 60 years and older. The collected data provided a rich database for relevant indicators, enabling us to evaluate the association between abdominal obesity and diabetes and further optimize our assessment indicators. Secondly, anthropometry and questionnaires are managed by the same well-trained research team to ensure the quality of the data. Therefore, in the absence of a more simple and advanced indicator of abdominal obesity, CVAI is expected to be a reliable indicator for visceral obesity assessment in the Chinese population and insulin resistance and diabetes in Asians (5, 34) to better reflect the adverse systemic effects of excessive visceral obesity. There are some limitations to our study. First of all, since this study was conducted in a single center and was a cross-sectional study, it was not possible to make causal inferences about the relationship between phenotypic indicators of abdominal obesity and diabetes, and the prediction could only provide some directions. Later, our cohort was needed to further verify the relationship between CVAI and DM. Secondly, without direct measurement of insulin resistance, we could not directly evaluate the relationship between CVAI, NC, and WC and insulin resistance. Third, the lack of availability of glucose data 2 h after meals may lead to underdiagnosis in some diabetic subjects. The main advantages of this study include the richness of correlation measures provided by the data set, and the large sample size allowing us to assess the various associations and predictive powers of a set of indices.

## 5. Conclusion

In general, for people aged 60 years and older, the risk of DM and other diseases can no longer be measured by traditional anthropometric indicators such as WC alone, while CVAI is consistent with the features of fat distribution in the older individual and shows superior discriminability for diabetes, which requires further prospective studies to verify our findings in external populations. To sum up, maintaining the balance of fat, muscle and bone is essential for maintaining metabolic homeostasis and health in the older individual.

## Data availability statement

The original contributions presented in the study are included in the article/Supplementary material, further inquiries can be directed to the corresponding authors.

## Ethics statement

The study protocol was approved by the Ethics Committee of Guangdong Pharmaceutical University, and all study participants provided written informed consent.

## Author contributions

XF, TS, and SW: conceptualization. XF and JW: methodology and software. XF and TS: data curation. TS and QC: project administration. ZW, LL, and YW: investigation. XF: writing original draft. XF, SW, TS, and QC: formal analysis. SPW did a lot of work on the experimental design and data processing analysis of the manuscript. After receiving the reviewers’ comments, she made constructive suggestions in the revised version of the manuscript and made important contributions to the paper attacking the problem. All authors contributed to the article and approved the submitted version.

## Funding

This research received external funding, including: Guangdong Province Medical Science and Technology Research Fund (No. 2019GCZX012); General Project of Guangzhou Basic and Applied Basic Research Project (No. 202201010545).

## Conflict of interest

The authors declare that the research was conducted in the absence of any commercial or financial relationships that could be construed as a potential conflict of interest.

## Publisher’s note

All claims expressed in this article are solely those of the authors and do not necessarily represent those of their affiliated organizations, or those of the publisher, the editors and the reviewers. Any product that may be evaluated in this article, or claim that may be made by its manufacturer, is not guaranteed or endorsed by the publisher.
