# Supplementary material for: Correlation analysis of anthropometric indices and type 2 diabetes mellitus in residents aged 60 years and older
Source: Front Public Health. 2023 Mar 29;11:1122509. doi: 10.3389/fpubh.2023.1122509 (PMC10095560; doi:10.3389/fpubh.2023.1122509)
Supplement: Supplementary file 1 [file Data_Sheet_2.PDF]

*Supplementary Material*

# Correlation Analysis of Abdominal Obesity Index and Type 2 Diabetes Mellitus in Residents Aged 60 Years and Above

**Xiaoyan Feng[1,2], Junyi Wang[1,2], Shan Wu[1,2], Zhihao Wang[1,2], Yuan Wei[1,2], Lvrong Li[1,2], Tianran Shen[1,2*], Qingsong Chen[1,2*]**

*** Correspondence:** Corresponding Author: Qingsong Chen: qingsongchen@aliyun.com;
Tianran Shen: shentrgz@163.com

## 1 Supplementary Data

[金山文档] DATE.xlsx

## 2 Sample size calculation

This study is a cross-sectional study. The sample calculation formula is as follows:

$$N = \frac{Z_{1-\alpha/2}^{2}\pi(1-\pi)}{\delta^{2}}$$

In the formula：N: Sample size; $Z_{1-\alpha/2}$: area under the corresponding standard normal distribution curve; π: Standard deviation of overall rate; δ: Allowable error.

Meet the following conditions：

①The prevalence rate of diabetes among the elderly in China is about 30.2%[10]; ②The allowable error is controlled within 10%, and the value is 2% to ensure the accuracy; ③α=0.05(bilateral)；④Considering the loss of follow-up rate of 20%；⑤Cluster sampling design effect (Deff) =2.

After calculation, at least 5, 164 people need to be included in this study. A total of 9519 participants were included in this study, meeting the sample requirements.

## 3 Supplementary Figures and Tables

For more information on Supplementary Material and for details on the different file types accepted, please see here.

## 3.1 Supplementary Figures

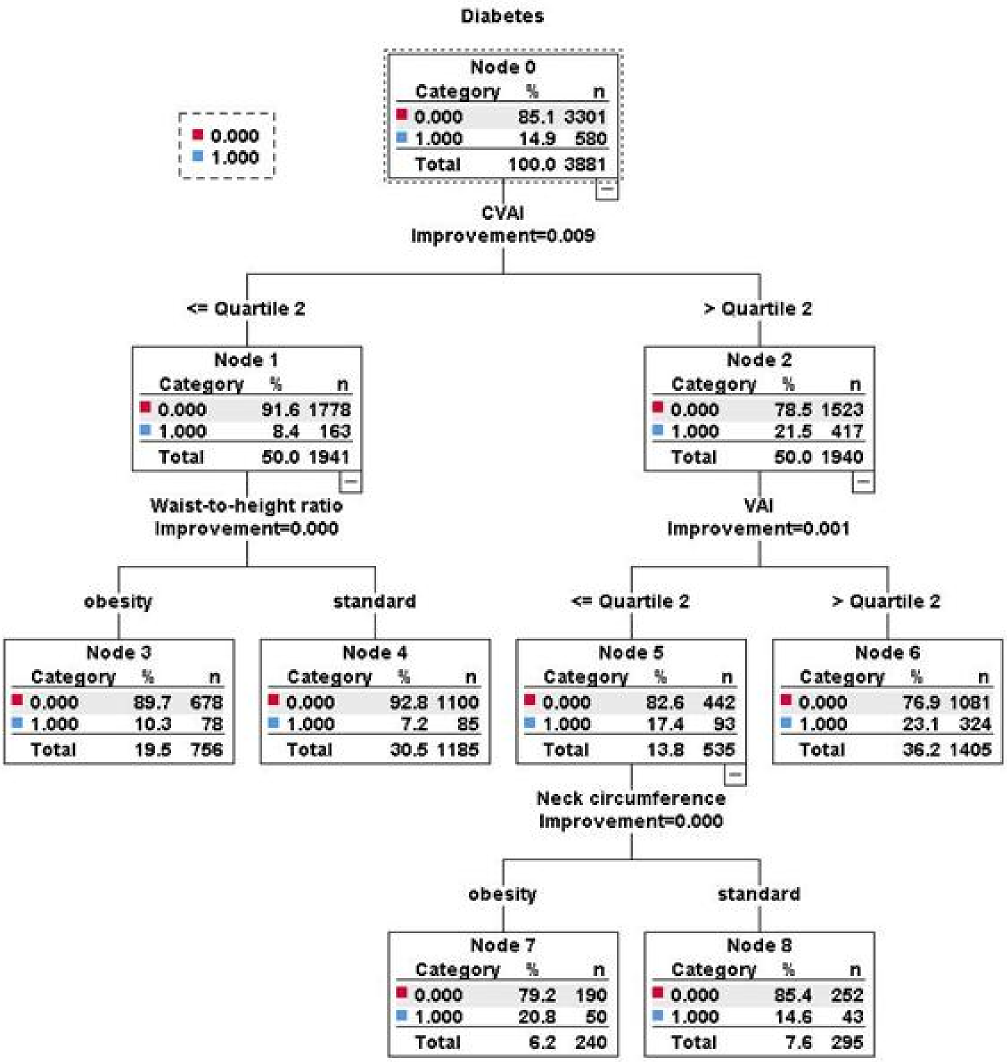


**Supplementary Figure 1. Male decision tree. Supplementary.** BMI, NC, WC, WHR, WHtR, VAI and CVAI were put into the Decision Tree model for analysis to help verify the results. Shows that in men, increased levels of NC, WHR and CVAI are associated with a higher prevalence of DM.

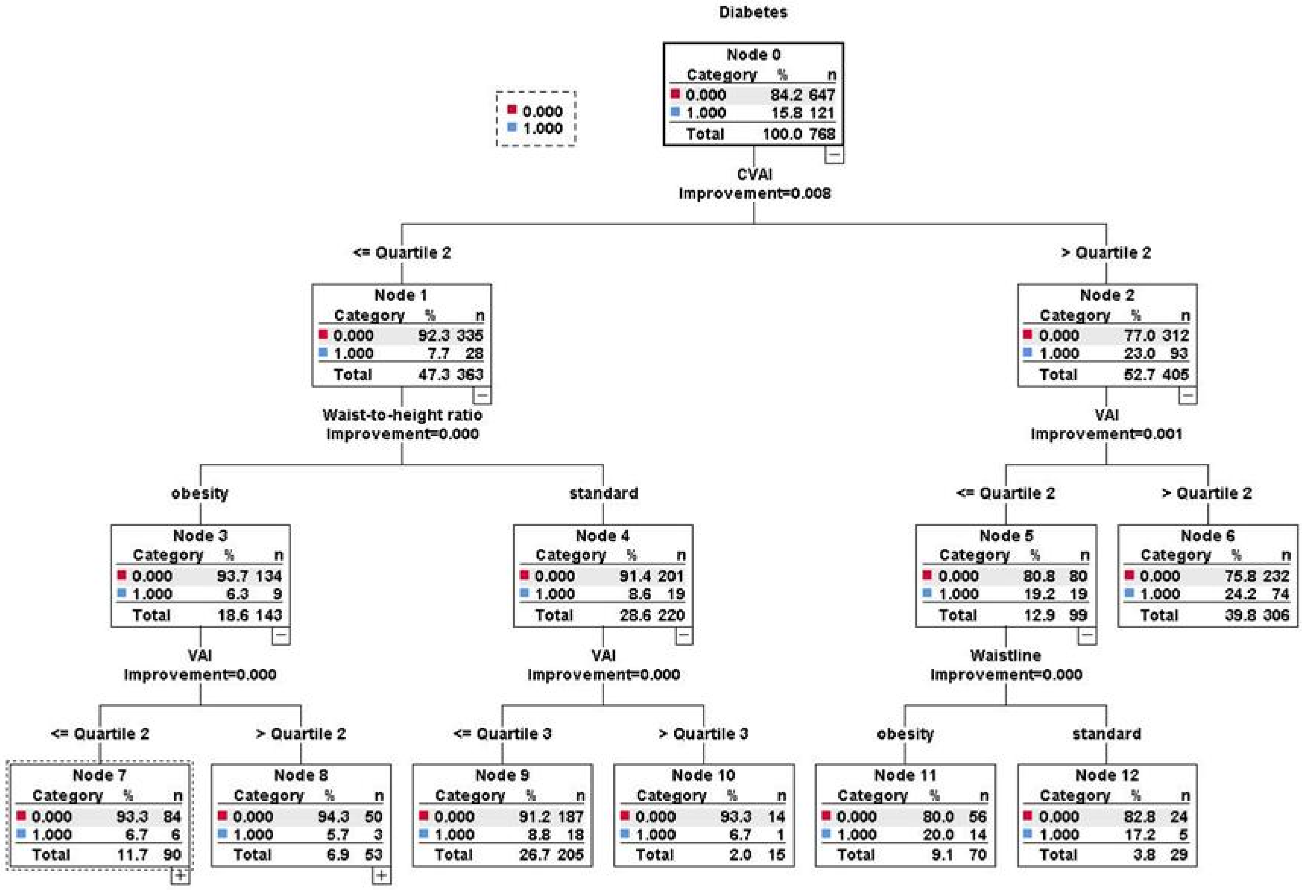


**Supplementary Figure 2. Decision tree for women.** BMI, NC, WC, WHR, WHtR, VAI and CVAI were put into the Decision Tree model for analysis to help verify the results. Shows that in women, increased levels of WC, WHtR, VAI and CVAI were associated with increased prevalence of DM.

## 3.2 Supplementary Tables

**Table S1 Correlations between obesity indicators (spearman correlation coefficient, rs)**

| | **BMI** | **NC** | **WC** | **WHR** | **WHtR** | **VAI** | **CVAI** | **LAP** |
|---|---|---|---|---|---|---|---|---|
| BMI | 1.000 | -231 | -0.105 | 0.415 | -0.263 | 0.107 | -0.279 | -0.056 |
| NC | | 1.000 | -0.495 | -0.058 | 0.459 | 0.044 | 0.019 | -0.030 |
| WC | | | 1.000 | -0.410 | -0.398 | 0.280 | -0.307 | -0.218 |
| WHR | | | | 1.000 | -0.255 | -0.058 | 0.030 | -0.410 |
| WHtR | | | | | 1.000 | 0.072 | -0.261 | -0.044 |
| VAI | | | | | | 1.000 | -0.177 | **-0.897** |
| CVAI | | | | | | | 1.000 | -0.059 |
| LAP | | | | | | | | 1.000 |

**Table S2 Multivariate analysis of anthropometric Indices and diabetes mellitus in the older individual**

| Variable | Men | | | Woman | | |
|---|---|---|---|---|---|---|
| | **OR** | **95% CI** | ***P*** | **OR** | **95% CI** | ***P*** |
| **NC** | 1.056 | 1.007-1.107 | 0.025 | 1.068 | 1.029-1.110 | 0.001 |
| **WC** | 0.899 | 0.830-0.974 | 0.009 | 0.999 | 0.972-1.026 | 0.916 |
| **WHR** | 1.061 | 1.033-1.090 | ＜0.001 | 1.040 | 1.022-1.058 | ＜0.001 |
| **WHtR** | 0.983 | 0.933-1.035 | 0.508 | 0.972 | 0.938-1.008 | 0.123 |
| **VAI** | 0.962 | 0.888-1.041 | 0.334 | 1.018 | 0.989-1.048 | 0.220 |
| **CVAI** | 1.033 | 1.014-1.053 | ＜0.001 | 1.011 | 1.003-1.019 | 0.008 |

* WHR and WHtR are based on this value ×100. Adjusted for age, education level, smoking, alcohol consumption, physical activity, BMI, diastolic blood pressure, systolic blood pressure and fasting blood glucose.

The definition of DM was based on registered diagnosis, abnormal fasting blood glucos, treatment with antidiabetic drugs and self-reported DM.

**Table S3 Multivariate analysis of anthropometric Indices and diabetes mellitus in the older individual**

| Variable | Men | | | Woman | | |
|---|---|---|---|---|---|---|
| | **OR** | **95% CI** | ***P*** | **OR** | **95% CI** | ***P*** |
| **NC** | 1.071 | 1.022-1.122 | 0.004 | 1.049 | 1.010-1.090 | 0.013 |
| **WC** | 0.929 | 0.863-1.000 | 0.051 | 1.003 | 0.977-1.030 | 0.837 |
| **WHR** | 1.064 | 1.036-1.093 | ＜0.001 | 1.031 | 1.013-1.049 | 0.001 |
| **WHtR** | 0.979 | 0.930-1.030 | 0.404 | 0.989 | 0.954-1.025 | 0.532 |
| **VAI** | 0.990 | 0.916-1.069 | 0.792 | 1.030 | 0.997-1.065 | 0.076 |
| **CVAI** | 1.020 | 1.003-1.038 | 0.019 | 1.004 | 0.996-1.012 | 0.374 |

* WHR and WHtR are based on this value ×100. Adjusted for age, education level, smoking, alcohol consumption, physical activity, BMI, diastolic blood pressure, systolic blood pressure and fasting blood glucose.
